# Excellent Thermoelectric Performance Realized in SnSe Materials via Chemical Bond Tailoring Using GeTe as Molecule Scissors

**DOI:** 10.1002/advs.78128

**Published:** 2026-09-30

**Authors:** Xing Yang, Wang‐Qi Bao, Zi‐Yuan Wang, Ze Li, Rong Li, Yi‐Xin Zhang, Jing Feng, Zhen‐Hua Ge, Li‐Dong Zhao

**Affiliations:** ^1^ Faculty of Materials Science and Engineering Kunming University of Science and Technology Kunming China; ^2^ National‐Local Joint Engineering Research Center For Technology of Advanced Metallic Solidification Forming and Equipment Kunming University of Science and Technology Kunming China; ^3^ School of Materials Science and Engineering Beihang University Beijing China

**Keywords:** Ag_2_Se precipitates, chemical bond tailoring, electrical transport properties, lattice thermal conductivity, polycrystalline SnSe

## Abstract

Tin selenide (SnSe) exhibits great potential in thermoelectrics, owing to the innocuity and intrinsic low thermal conductivity. However, the poor electrical transport properties seriously limit the performance improvement and practical application. Herein, we report that GeTe alloying in Ag‐doped SnSe acts as a “molecular scissor” to tailor chemical bond, inducing Ag_2_Se precipitation. This simultaneously enhances the Seebeck coefficient (632 µV K^−1^ at 523 K for the A‐SnSe + 0.5 wt.% GeTe sample) via controlling carrier concentration and suppresses the lattice thermal conductivity to an ultralow 0.25 W m^−1^ K^−1^ at 823 K through multi‐scale phonon scattering. This synergistic optimization yields a high *ZT* of 1.75 at 823 K and an impressive average *ZT* of 0.96 (623–823 K). This chemical bond tailoring strategy, validated by defect formation energy and DOS calculations, provides a general approach for improving thermoelectric performance.

## Graphical abstract

The GeTe acting as “molecule scissors” can tailor chemical bond, the breaking of Ag‐Se bonds and the formation of Ge‐Se bonds occur in the A‐SnSe matrix, along with the formation of Ag_2_Se precipitates. This process helps to optimize the thermoelectric properties, and also improves its mechanical properties to a certain extent.

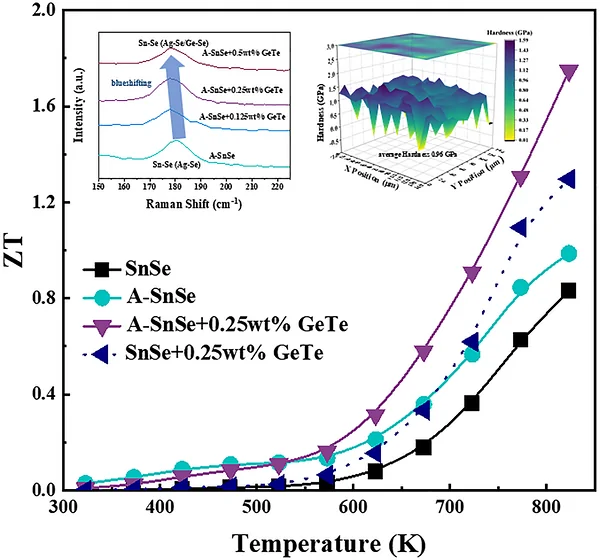

## 1 Introduction

Thermoelectric materials can transform heat into electricity and electricity into heat through direct energy conversion [1]. Especially, the fossil energy consumption is skyrocketing today, and most of the energy is discharged into the environment as waste heat [2, 3]; therefore, thermoelectric materials that can effectively make use of waste heat are one of the most promising materials [4]. The dimensionless thermoelectric figure of merit (*ZT*), expressed as *(σS^2^T)/κ*, serves as the primary metric for evaluating material thermoelectric performance, where *σ* denotes electrical conductivity, *S* represents the Seebeck coefficient, *T* is absolute temperature, and *κ* signifies total thermal conductivity. Enhanced *ZT* values are achieved through optimization of either electrical transport properties (high *σS^2^*) or thermal insulation characteristics (low *κ*) [5, 6, 7]. Advanced thermoelectric optimization strategies currently focus on three primary approaches: band structure engineering [8, 9, 10], nanoscale architectural design [11, 12, 13], and controlled grain boundary modification [14, 15, 16, 17, 18, 19, 20, 21]. These synergistic techniques facilitate the cooperative optimization of traditionally interdependent thermoelectric parameters (electrical conductivity, Seebeck coefficient, and thermal conductivity) through precise control of carrier concentration. This decoupling approach enables simultaneous enhancement of the power factor while suppressing lattice thermal conductivity, achieving unprecedented performance [22].

SnSe materials demonstrate exceptional potential for thermoelectric applications due to their naturally low thermal conductivity, a property arising from their unique layered crystalline architecture [11, 23, 24]. Besides, the low toxic and earth‐abundant raw materials that can synthesize the SnSe is also a key factor. SnSe crystals has garnered significant research interest owing to exceptional thermoelectric properties, particularly the reported *ZT* value reaching 2.6 at 923 K [25]. Nevertheless, crystalline SnSe materials face substantial application barriers due to their inadequate mechanical strength and complicated synthesis procedures [26]; thus, the improvement of thermoelectric performance is urgent for SnSe polycrystals [27]. Focusing on the problems, many works have been done [28]. Based on the intrinsic low thermal conductivity, the optimizing strategies are mainly developed from electrical transport properties [29]. The improvement of electrical conductivity for SnSe materials is due to enhanced hole carrier induced by the alkalis metals [30, 31, 32], Ag [33, 34], Ga [35], and Cu [36, 37] doping, which has been proven that these are effective and successful and this is also the optimization strategies for most works. Another approach to enhancing electrical transport properties involves reducing the carrier concentration to obtain a high Seebeck coefficient [38]. Following this strategy, the introduction of Ge into the SnSe matrix has been shown to improve the Seebeck coefficient by 18% compared to undoped samples [14, 39]. Consequently, this optimization leads to better thermoelectric performance [40]. However, since the undoped SnSe sample exhibits inherently poor thermoelectric properties, developing new strategies to further enhance the performance of SnSe‐based materials remains an urgent priority.

In this work, we demonstrate a novel strategy to enhance the thermoelectric performance of p‐type polycrystalline SnSe materials by chemical bond talioring to optimize the Seebeck coefficient. Building on our previous study of 1 mol% Ag‐doped SnSe (denoted as A‐SnSe) [41], we further introduce GeTe into the A‐SnSe matrix to optimize electrical transport properties through Seebeck coefficient modulation. Notably, the incorporation of GeTe acts as “molecule scissors”, facilitating the formation of Ge‐Se bonds while cleaving Ag‐Se bonds in the A‐SnSe matrix. This bond reorganization leads to the precipitation of Ag_2_Se, significantly altering the electrical transport properties, as shown in Figure 1a; and with the increase of GeTe content, the amount of Ag_2_Se precipitates increases, and the increased defect formation energy of GeTe‐doped samples also confirms that. Furthermore, density functional theory (DFT) calculations reveal that the widened energy gap suppresses carrier transport, leading to an enhanced Seebeck coefficient. The A‐SnSe + 0.5 wt.% GeTe sample achieves a peak *S* value of 632 µV K^−1^ at 523 K. This improvement in the Seebeck coefficient contributes to a significantly enhanced power factor, reaching 712 µW m^−1^ K^−2^ at 823 K for the A‐SnSe + 0.25 wt.% GeTe sample (Figure 1b). Concurrently, the introduction of GeTe reduces lattice thermal conductivity through two key mechanisms: increased grain boundary scattering and mass fluctuation between Sn‐Ge and Se‐Te atoms. As a result, an ultralow *κ*_l_ value of 0.25 W m^−1^ K^−1^ is attained at 823 K for the A‐SnSe + 0.25 wt.% GeTe sample. The synergistic optimization of electrical and thermal transport properties yields a high *ZT* value of 1.75 at 823 K and a competitive average *ZT* value of 0.96 from 623 to 823 K. Additionally, GeTe doping enhances mechanical properties via solid‐solution strengthening and Ag_2_Se precipitates (Figure 1c). These findings demonstrate the potential of GeTe‐modified SnSe materials for medium‐temperature power generation applications.

**FIGURE 1:**
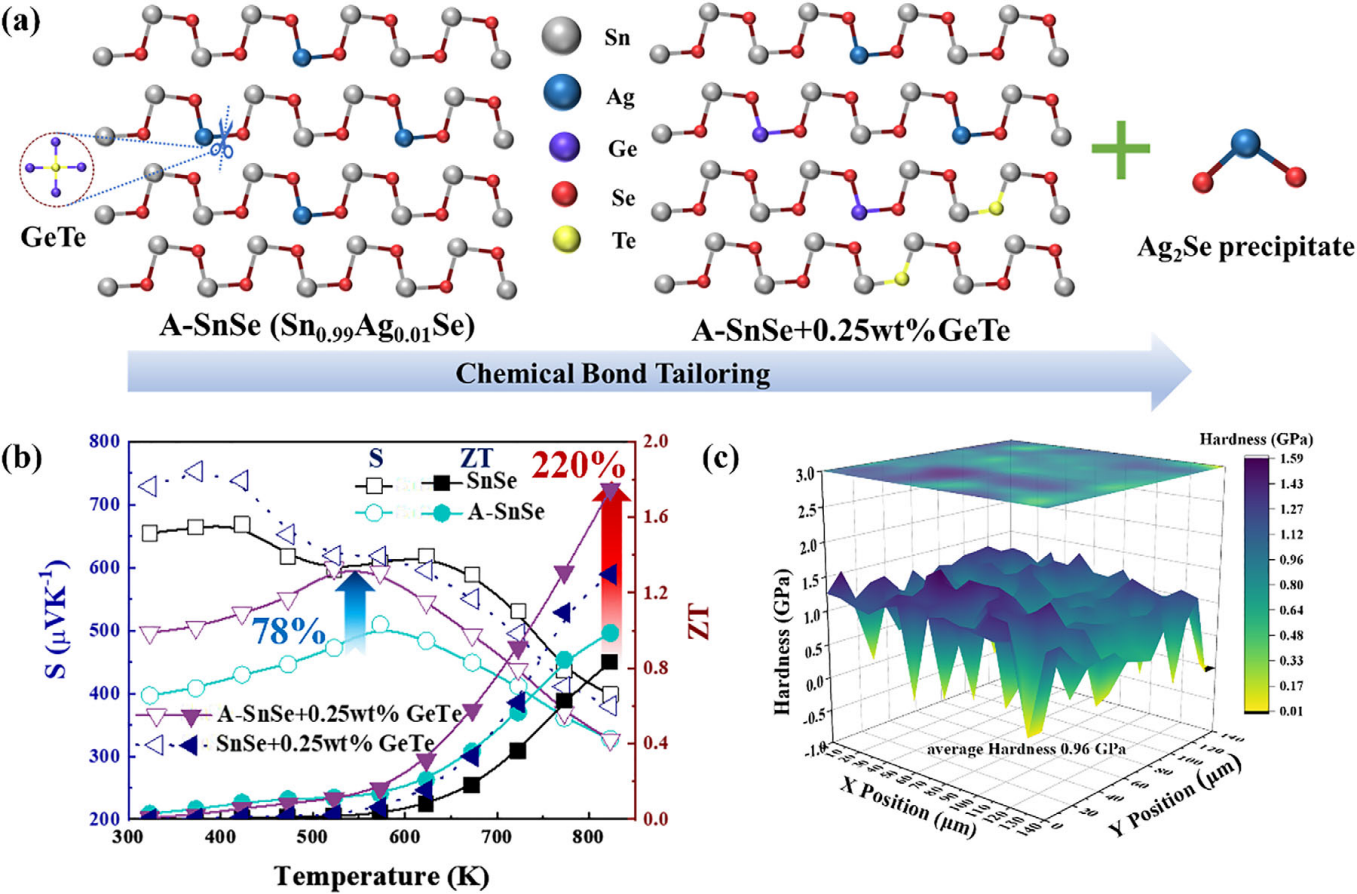
(a) Schematic diagram of the chemical bond tailoring SnSe/Ag_2_Se composites, (b) the Seebeck coefficient and ZT values for the pristine SnSe sample, Sn_0.99_Ag_0.01_Se sample, and A‐SnSe + 0.25 wt.% GeTe sample, (c) the distribution of hardness for the A‐SnSe + 0.25 wt.% GeTe sample.

## 2 Experimental Sections

The pristine SnSe and A‐SnSe + x wt.% GeTe (x = 0, 0.125, 0.25, and 0.5) samples were prepared using a two‐step process combining melting and spark plasma sintering (SPS). High‐purity elemental powders (Ag, Sn, Se) and GeTe compound were precisely weighed according to stoichiometric ratios. The raw materials were loaded into a high‐purity quartz tube, which was then evacuated to 10^−4^ Pa and flame‐sealed. For additional safety, the sealed tube was placed inside a larger quartz tube and again evacuated to 10^−4^ Pa before sealing. The double‐encapsulated ampoule was heated in a vertical tube furnace using the following temperature profile: (1) heating to 1223 K at a controlled rate of 2 K/min, (2) holding at 1223 K for 24 h for homogenization, and (3) furnace cooling to room temperature at 1 K/min. The resulting ingots were manually ground in an argon‐filled glove box to prevent oxidation. The powders were then consolidated into dense pellets using SPS at 723 K under 50 MPa uniaxial pressure for 5 min.

Phase identification and structural analysis were conducted using a Rigaku MiniFlex600 X‐ray diffractometer equipped with Cu Kα radiation (λ = 1.5406 Å). The Raman spectra is measured under 325 nm. For microstructural characterization, we employed: (i) scanning electron microscopy (Zeiss Sigma300), (ii) high‐resolution transmission electron microscopy (Thermo Fisher Talos F200X), and (iii) wavelength‐dispersive X‐ray spectroscopy (Shimadzu EPMA‐1720H). The temperature‐dependent electrical conductivity and Seebeck coefficient were measured simultaneously under helium atmosphere using an Ulvac ZEM‐3 system. Thermal transport properties were determined through laser flash analysis (Netzsch LFA‐457) for thermal diffusivity measurements, with sample densities measured via the Archimedes method using deionized water. Mechanical properties were evaluated through nanoindentation tests using an iMicro system, with hardness and Young's modulus values obtained at an applied force of 20 mN to ensure minimal substrate effects.

First‐principles DFT calculations were performed using VASP with PAW pseudopotentials and a 400 eV plane‐wave cutoff. The PBE‐GGA functional treated exchange‐correlation effects. A 4 × 6 × 6 k‐mesh ensured convergence. Structural relaxations continued until forces reached 0.01 eV/Å and energy changes fell below 10^−6^ eV. A 2 × 3 × 3 supercell modeled doping effects. The ATAT package generated 50 SQS configurations, with the most stable (<5 meV/atom) selected for analysis. Post‐processing included band unfolding and Born charge calculations to examine modified electronic states and bonding.

## 3 Results and Discussion

The crystal structure of all synthesized SnSe‐based samples was systematically investigated using X‐ray diffraction (XRD). As shown in Figure 2a, all diffraction patterns for all samples are consistent with the standard PDF card (PDF#48‐1224). As shown in Figure 2b, when Ag element is introduced in the SnSe matrix, the XRD patterns shift to the higher angles due to the smaller ionic radii of Ag^+^ (0.115 nm) in comparison with Sn^2+^ (0.118 nm). However, when GeTe is further introduced in the A‐SnSe matrix, the XRD patterns shift to the lower angles. Considering the ionic radii difference between Sn^2+^, Ge^2+^ (0.113 nm), Ag^+^, Se^2−^ (0.198 nm), and Te^2−^ (0.221 nm), when Ge^2+^ and Te^2−^ enter the Sn^2+^ and Se^2−^, respectively, which can cause the lattice expansion, therefore, the XRD patterns should have continued to shift at a lower angle, this is unusual. And from the measured XRD patterns results, as the GeTe content increases, the XRD patterns gradually shift to the higher angles.

**FIGURE 2:**
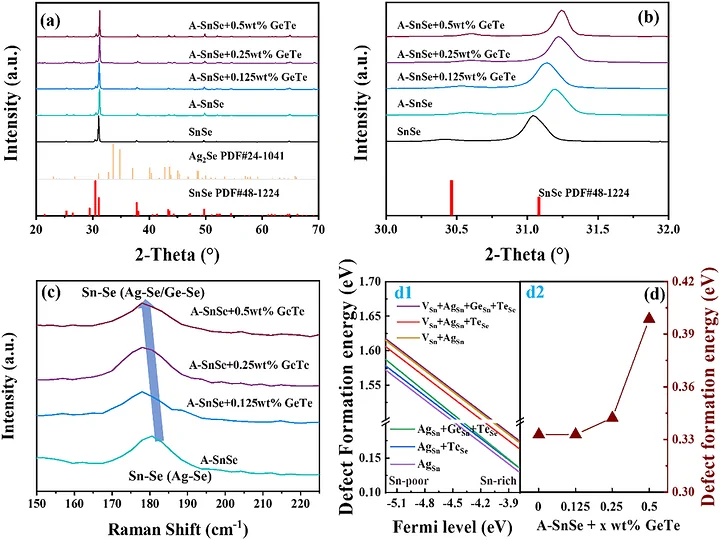
(a) XRD patterns, (b) enlarged XRD patterns in 2‐Theta range of 30–32°, (c) Raman spectra, and (d1) the defect formation energy of different sites, (d2) the value of defect formation energy for *Ag_Sn_* sites with different GeTe content.

To make sense of this anomalous XRD peak shifting behavior, the formation energy of different defects is depicted in Figure 2d1. Based on the calculation results of defect formation energy, when GeTe is further introduced into the A‐SnSe matrix, the formation energy of *V_Sn_ + Ag_Sn_ + Ge_Sn_ + Te_Se_* defects is higher than that of *V_Sn_ + Ag_Sn_ + Te_Se_* and *V_Sn_ + Ag_Sn_* defects, suggesting that the formation energy of *Ag_Sn_* defect increases, resulting in the Ag^+^ segregation. Moreover, combined with the results of formation energy for *Ag_Sn_* as the GeTe content increases, as shown in Figure 2d2, the formation energy of *Ag_Sn_* increases, indicating a fundamental change in the bonding environment. This phenomenon can be attributed to the unique role of GeTe as “molecule scissors” that chemically modifies the lattice through bond reorganization: cleavage of existing Ag‐Se bonds and concurrent formation of new Ge‐Se bonds, ultimately leading to Ag_2_Se precipitation. Figure 2c presents the Raman spectra of A‐SnSe + x wt.% GeTe (x = 0, 0.125, 0.25, and 0.5) bulk samples. With increasing GeTe content, a distinct blueshift is observed in the Raman spectra. This phenomenon can be attributed to the formation of Ge‐Se bonds, which exhibit a shorter bond length compared to the original Sn‐Se and Ag‐Se bonds. According to the harmonic oscillator model, the shorter Ge‐Se bond corresponds to a higher force constant, thereby leading to an increase in vibrational frequency. This bond‐length‐induced stiffening effect results in the systematic blueshift observed in the Raman spectra. Elemental distribution analysis was performed using electron probe microanalysis (EPMA), as presented in Figure S1. The EPMA mapping reveals homogeneous dispersion of all constituent elements (Sn, Ag, Ge, Se, and Te) throughout the SnSe matrix, while clearly identifying the presence of Ag_2_Se precipitates, this also confirms that the Ag^+^ segregation is existed as the Ag_2_Se precipitates. This observation provides direct experimental evidence supporting the calculated increase in defect formation energy. Combined with the unusual XRD pattern shift, as the GeTe content increases in the A‐SnSe matrix, the amount of Ag_2_Se precipitate increases. The Ag_2_Se precipitate second phase from the tin selenide lattice causes the lattice to shrink, therefore, the XRD patterns shifted to a higher angle.

Microstructural characterization reveals the significant impact of GeTe incorporation on the SnSe matrix, as shown in Figure S2. While all compositions maintain the intrinsic layered structure of SnSe, distinct grain evolution is observed. Initial Ag doping facilitates grain growth through enhanced atomic mobility, whereas subsequent GeTe addition induces progressive grain refinement. This size reduction stems from the pinning effect of Ag_2_Se precipitates at grain boundaries, which effectively inhibit grain coarsening. Correspondingly, density measurements (Figure S2f) demonstrate a systematic increase with GeTe content, attributable to improved particle packing efficiency and reduced porosity resulting from the finer grain structure. These findings collectively demonstrate that GeTe incorporation preserves the fundamental SnSe layered architecture while enabling controlled grain refinement and enhanced densification through precipitation‐mediated microstructural engineering.

To further investigate the microstructure of the A‐SnSe + 0.25 wt.% GeTe sample, HRTEM analysis was performed. As shown in Figure 3a, the different contrasts can be seen from the brightfield phase diagram. Therefore, the EDS‐mapping is measured, the results are shown in Figure 3f–j. The elements, Sn, Se, Ag, Ge, and Te, are evenly distributed in the SnSe matrix. Obviously, the Ag precipitate exists in the SnSe matrix. Figure 3b is the enlarged figure of Figure 3a. From the high‐resolution image (Figure 3c), there are two different lattice fringes. According to the SAED images of the areas (Figure 3d,e), the SnSe phase is shown in Figure 3e, the Ag_2_Se phase is shown in Figure 3d, and the right part is the SnSe with the direction of [1 0 1] and the left part is the Ag_2_Se with the direction of [2 1 0]. To further analyze the A‐SnSe + 0.25 wt.% GeTe sample by TEM, as shown in Figure S4. The high‐resolution image of the selected area in Figure 6a is shown in Figure S3b. From Figure S3c, it can be seen that the high‐density dislocations exist the SnSe matrix in the enlarged area of Figure S3b. The GPA results from Figure S3b are shown in Figure S3d,e, which can be obviously observed that the stress concentration regions are present in dislocations, this is conducive to a reduction in the lattice thermal conductivity.

**FIGURE 3:**
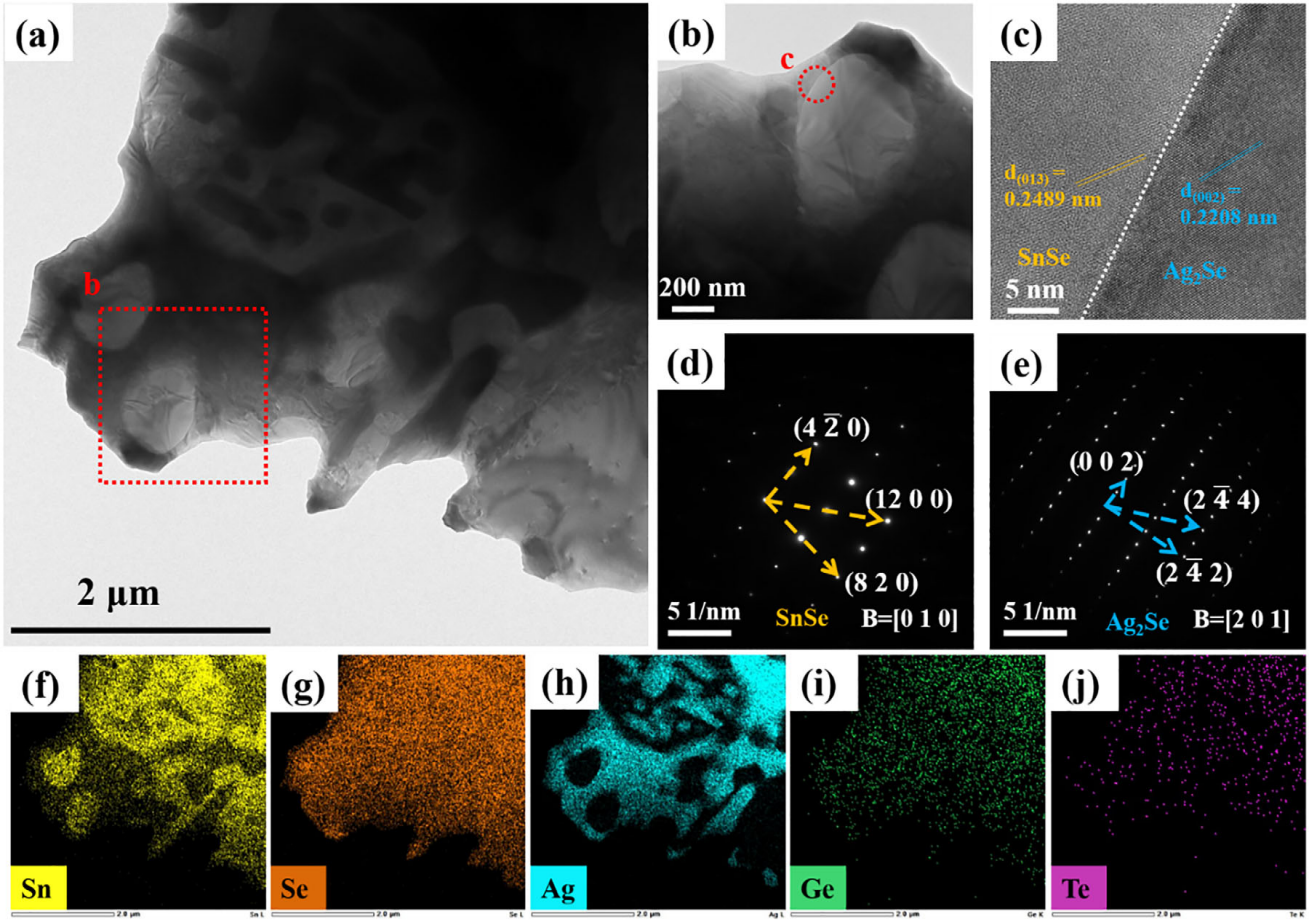
TEM information for the pristine SnSe and A‐SnSe + 0.25 wt.% GeTe sample (a‐b) low magnified images, (c) high magnified images, (d, e) the corresponding FFT image of (c), (f–j) the EDS‐mapping results of (a) and the elements of Sn, Se, Ag, Ge, and Te, respectively.

The temperature‐dependent electrical transport properties of pristine SnSe and A‐SnSe + x wt.% GeTe (x = 0, 0.125, 0.25, and 0.5) bulk samples, measured along the direction parallel to the SPS pressure axis, are presented in Figure 4. Figure 4a demonstrates that GeTe incorporation into the SnSe matrix leads to a significant reduction in electrical conductivity. This phenomenon primarily originates from the chemical bond tailoring effect induced by GeTe doping, which promotes the segregation of Ag from the SnSe lattice to form Ag_2_Se secondary phases. The possible defect reaction equations are shown below.

$$GeTe\overset{Sn_{0.99}Ag_{0.01}Se}{\to }aGe_{Ag}^{\cdot }+\left(1-a\right)Ge_{Sn}^{\times }+Te_{Se}^{\times }+0.5aAg_{2}Se+e^{\prime }$$

**FIGURE 4:**
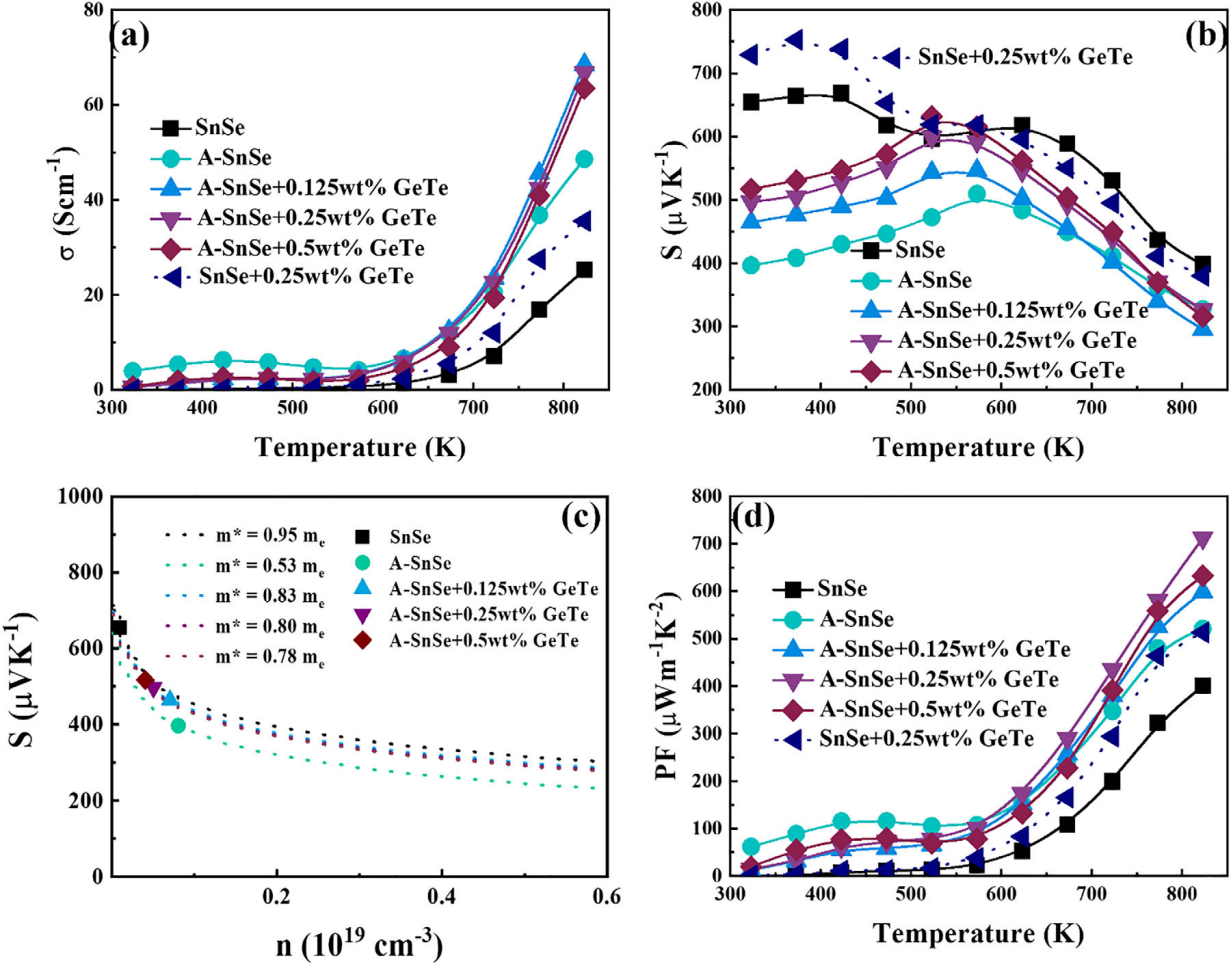
Temperature dependence of the electrical transport properties for the pristine SnSe and A‐SnSe + x wt.% GeTe (x = 0, 0.125, 0.25, and 0.5) bulk samples (a) electrical conductivity (*σ*), (b) Seebeck coefficient (*S*), (c) absolute values of *S* as a function of carrier concentration at room temperature, and (d) power factor (*PF*).

With the formation of Ag_2_Se precipitates, the electron carriers are released; resulting in a decrease of hole carrier concentration in the SnSe matrix. Furthermore, the substantial electronegativity differences between Ge‐Sn (Δχ = 0.3) and Te‐Se (Δχ = 0.1) pairs may create additional charge carrier scattering centers, further contributing to the conductivity reduction. The observed monotonic decrease in electrical conductivity with increasing GeTe content strongly correlates with enhanced Ag_2_Se precipitates, as confirmed by our XRD and TEM analyses (Figures 2 and 3). These results clearly demonstrate that GeTe acts as an effective ‘molecule scissors’ that modifies chemical bonding configurations, thereby controlling the precipitation behavior of Ag_2_Se secondary phases. Besides, Ag_2_Se shows an n‐type semiconductor, playing a role in inhibiting the increase in electrical conductivity. Compared to pristine SnSe sample, the GeTe single‐doped sample (SnSe + 0.25 wt.% GeTe) shows a marginal increase in electrical conductivity. However, this value remains lower than that of (Ag, GeTe) co‐doped samples, as confirmed in Figure 4a. This difference arises because a fraction of Ag atoms successfully incorporates into the SnSe lattice in the co‐doped samples, enhancing carrier concentration. The temperature‐dependent electrical conductivity exhibits a complex three‐stage behavior: first, an initial increase due to enhanced carrier mobility with rising thermal energy; second, a subsequent decrease caused by intensified grain boundary scattering at intermediate temperatures, and third, a final upswing at elevated temperatures resulting from thermal excitation of charge carriers. Notably, the A‐SnSe + 0.125 wt.% GeTe sample achieves an exceptional maximum electrical conductivity (*σ*) of 68.5 S cm^−^^1^ at 773 K, representing a significant improvement over undoped SnSe sample.

To elucidate the mechanism behind the conductivity changes induced by GeTe doping in the SnSe matrix, the density functional theory (DFT) calculations using the PBE functional was performed, as presented in Figure S4. The computational results reveal distinct electronic structure modifications depending on the dopant species. Figure S4b demonstrates that Ag incorporation into the SnSe lattice reduces the band energy, thereby facilitating carrier transport and consequently enhancing electrical conductivity in Ag‐doped samples. In contrast, Figure S4c shows that GeTe doping increases the band energy of A‐SnSe sample, creating a potential barrier that impedes carrier mobility and leads to reduced electrical conductivity. This phenomenon primarily occurs because GeTe doping promotes the segregation of Ag from the A‐SnSe matrix, forming Ag_2_Se precipitates that decrease the effective hole carrier concentration.

All samples exhibit positive Seebeck coefficients, confirming their p‐type semiconducting behavior, as shown in Figure 4b. When GeTe is introduced in the A‐SnSe matrix, the Seebeck coefficient increases, and with the increase of GeTe content, the Seebeck coefficient gradually increases. To reveal the reasons for the increase in Seebeck coefficient, the Single Parabolic Band (SPB) model is used to analysis the relationship between Seebeck coefficient, carrier concentration, and effective mass, which is based on the Matt law [42]. From the following formula:

$$S=\frac{8\pi^{2}\kappa_{B}^{2}}{3eh^{2}}m^{*}T{\left(\frac{\pi }{3n}\right)}^{2/3}$$

It is clear that the Seebeck coefficient is directly proportional to the effective mass and inversely proportional to the carrier concentration. As shown in Figure 4c, When GeTe is introduced in the A‐SnSe matrix, the carrier concentration decreases, and as the increase of GeTe content, the carrier concentration decreases gradually. The decreased carrier concentration is directly related to the increase in bandgap; the increased bandgap can suppress the carrier transport. Combined with SPB model, when GeTe enter the A‐SnSe matrix, the effective mass increases, resulting in an increase of the Seebeck coefficient. The temperature evolution of the Seebeck coefficient reveals characteristic non‐monotonic behavior, with an initial increase followed by a maximum at 523–573 K and subsequent decrease at higher temperatures. This feature originates from the onset of bipolar conduction, where thermal excitation of minority carriers becomes significant, the phenomenon is well‐documented in similar chalcogenide systems [32, 43, 44]. Furthermore, Ge doping in SnSe enhances the Seebeck coefficient through favorable band alignment effects [45]. Notably, a systematic shift of the peak Seebeck coefficient to lower temperatures with increasing GeTe content is observed. This phenomenon likely arises from band widening effects that promote minority carrier scattering. When GeTe is introduced, the Seebeck coefficient increases from 397 µV K^−1^ (A‐SnSe sample) to 517 µV K^−1^ (A‐SnSe + 0.5 wt.% GeTe sample), directly corresponding to the controlled reduction in carrier concentration due to the formed Ag_2_Se precipitates by tailoring chemical bond. Owing to the decreased carrier concentration and band alignment, the A‐SnSe + 0.5 wt.% GeTe sample achieves a remarkable peak *S* value of 632 µV K^−^^1^ at 523 K. This substantial improvement in the Seebeck coefficient, while maintaining reasonable electrical conductivity, leads to a significantly enhancement in power factors. Ultimately, the A‐SnSe + 0.25 wt.% GeTe sample reaches a peak power factor of 712 µW m^−1^ K^−2^ at 823 K, representing a 78% enhancement over pristine SnSe sample, as shown in Figure 4d. This breakthrough performance demonstrates the effectiveness of GeTe doping in optimizing the thermoelectric properties of SnSe‐based materials.

Figure 5 presents the temperature‐dependent thermal transport properties of pristine SnSe and A‐SnSe + x wt.% GeTe (x = 0, 0.125, 0.25, 0.5) bulk samples. The total thermal conductivity (*κ*_tot_) for all compositions shows a characteristic decreasing trend with temperature (Figure 5a), interrupted by a notable upturn above ∼723 K. This phenomenon arises from the increasing contribution of electronic thermal conductivity (*κ*_ele_) at elevated temperatures, as evidenced in Figure 5c. Compared to pristine SnSe sample, Ag‐doped samples exhibit higher *κ*_tot_ values. However, subsequent GeTe incorporation effectively suppresses *κ*_tot_ through enhanced phonon scattering. Since lattice thermal conductivity (*κ*_lat_) dominates the total thermal conductivity, the analysis on *κ*_lat_ to understand the observed variations is focused. Figure 5b reveals that Ag doping increases the lattice thermal conductivity of SnSe, attributable to grain growth during the doping process. However, subsequent GeTe incorporation effectively reduces the lattice thermal conductivity through multiple phonons scattering mechanisms. It is clear that as GeTe content increases, the lattice thermal conductivity shows a decreasing trend overall. The formation of Ag_2_Se precipitates creates smaller grains with higher boundary density, significantly enhancing boundary scattering of phonons. Furthermore, the introduced dislocations and point defects provide additional scattering centers, while the mass contrast between Sn‐Ge and Se‐Te atoms induces strong point defect scattering. Notably, the lattice thermal conductivity for the high content of GeTe sample (A‐SnSe + 0.5 wt.% GeTe sample) is higher than that of the low content sample (A‐SnSe + 0.125 wt.% GeTe sample and A‐SnSe + 0.25 wt.% GeTe sample), the possible reason is that when GeTe exceeds 0.25 wt.%, the amount of Ag_2_Se precipitates increases, at elevated temperatures, these fine precipitates are prone to Ostwald ripening, coarsening into larger particles. These synergistic effects lead to a progressive reduction in lattice thermal conductivity with increasing GeTe content, culminating in an ultralow *κ*_lat_ value of 0.25 W m^−1^ K^−1^ at 823 K for the A‐SnSe + 0.25 wt.% GeTe sample. The Debye‐Callaway model analysis (Figure 5d) quantitatively demonstrates the distinct scattering contributions: grain boundaries primarily scatter low‐frequency phonons, while dislocations, point defects, and precipitates effectively scatter mid‐ to high‐frequency phonons. This hierarchical phonon scattering approach enables broad‐spectrum thermal conductivity suppression across the entire phonon frequency range.

**FIGURE 5:**
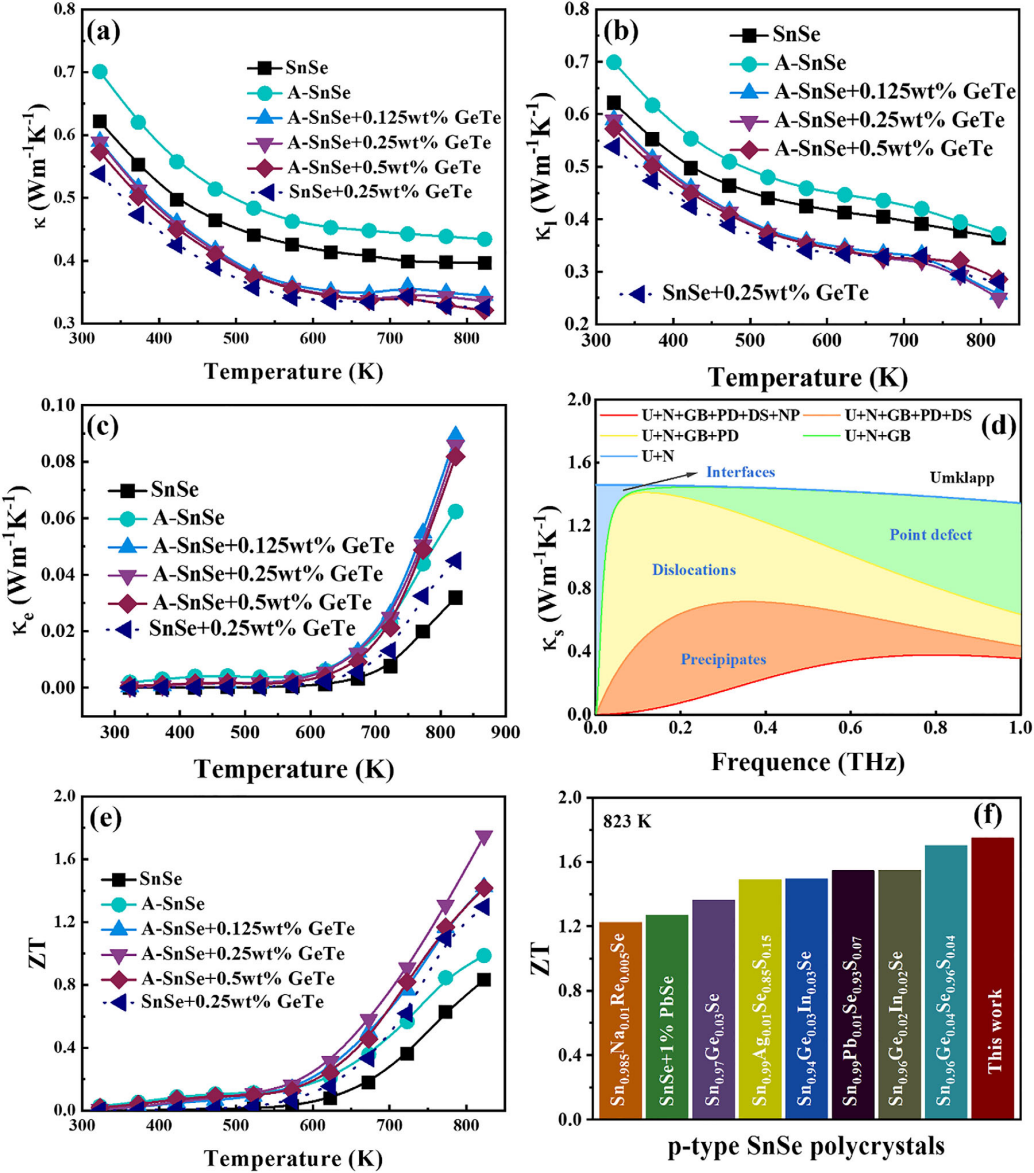
Temperature dependence of thermal transport properties and *ZT* values for the pristine SnSe and A‐SnSe + x wt.% GeTe (x = 0, 0.125, 0.25 and 0.5) bulk samples (a) total thermal conductivity (*κ*_tot_), (b) lattice thermal conductivity (*κ*_lat_), (c) electron thermal conductivity (*κ*_ele_), (d) calculated spectral lattice thermal conductivity (*κ*_s_) at 300 K using the Debye‐Callaway model, (e) ZT values, and (f) comparison with the reported works about *ZT* peak values at 823 K for p‐type polycrystalline SnSe materials.

The synergistic optimization of electrical and thermal transport properties in these GeTe‐doped SnSe materials leads to remarkable thermoelectric performance enhancement. The A‐SnSe + 0.25 wt.% GeTe composition achieves a peak *ZT* value of 1.75 at 823 K and an exceptional average ZT of 0.96 across the 623–823 K temperature range; these values represent 2.2‐ and 2.4‐times improvements over pristine SnSe sample, respectively; as shown in Figure 5e. From the Figures S5 and S6, the A‐SnSe + 0.25 wt.% GeTe is reprepared and performed cyclic test, it is clear that the phase structure of SnSe is maintained after 3 cycle tests, and the thermoelectric performance remains stable within errors, which confirm that the good reproducibility of our synthesis and measurement methods. Among state‐of‐the‐art p‐type polycrystalline SnSe materials [45, 46, 47, 48, 49, 50, 51, 52], our results realized by chemical bond tailoring demonstrate superior performance, particularly in the medium‐temperature range ideal for power generation applications (Figure 5f). The achieved performance metrics suggest these materials are promising candidates for practical thermoelectric energy conversion devices.

The distribution of hardness and modulus is shown in Figure 6. Compared with the pristine SnSe sample, when the Ag element enters the SnSe matrix, the hardness and modulus increase due to the solution strengthening, and when GeTe enters the A‐SnSe matrix, the hardness and modulus increase due to the solution strengthening and diffusion strengthening caused by Ag_2_Se precipitates, which provides a positive preliminary foundation for the subsequent research on device integration.

**FIGURE 6:**
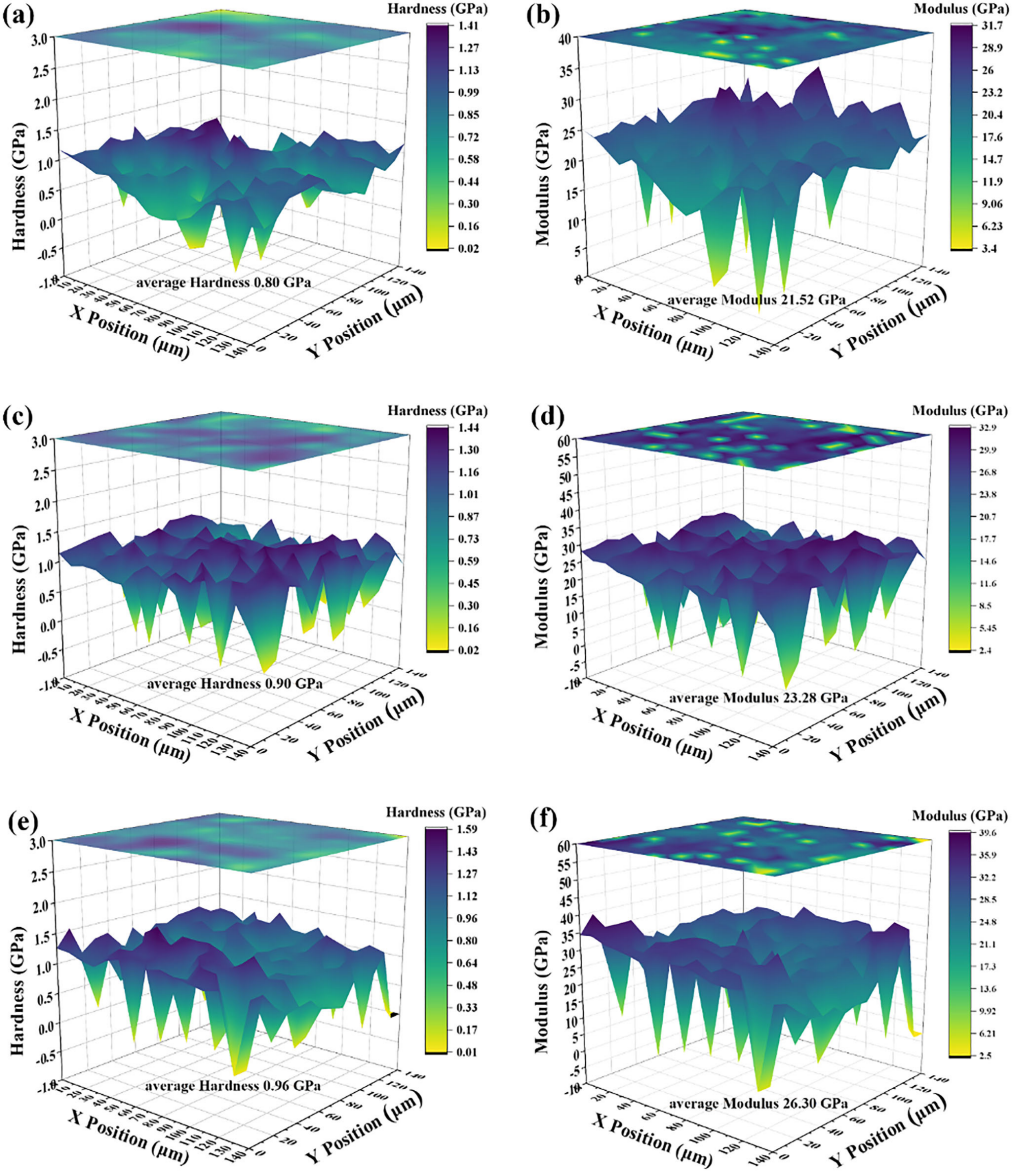
The distribution of hardness and modulus for (a) and (b) the pristine SnSe sample, (c) and (d) A‐SnSe (Sn_0.99_Ag_0.01_Se) sample, (e) and (f) A‐SnSe + 0.25 wt.% GeTe sample.

## 4 Conclusions

In this work, (Ag, GeTe) co‐doped polycrystalline SnSe bulk samples were synthesized via melting followed by spark plasma sintering (SPS). GeTe acts as “molecule scissors” to tailor chemical bonds in the A‐SnSe matrix, inducing the segregation of Ag as Ag_2_Se secondary phases. As the GeTe content increases, the Ag_2_Se precipitation becomes more pronounced, leading to two critical effects that decouple the thermal and electrical transport properties: (1) The reduction in hole carrier concentration due to Ag_2_Se precipitation significantly enhances the Seebeck coefficient, yielding a peak power factor (*PF*) of 712 µW m^−1^ K^−2^ at 823 K for the A‐SnSe + 0.25 wt.% GeTe sample. (2) The Ag_2_Se precipitates increase grain boundary density, intensifying phonon scattering. Coupled with mass fluctuation between Ge‐Sn and Te‐Se, this results in an ultralow lattice thermal conductivity (0.25 W m^−1^ K^−1^ at 823 K). These synergistic effects enable a high *ZT* (1.75 at 823 K) and an exceptional average *ZT* (*ZT*_ave_ = 0.96, 623–823 K), outperforming most reported p‐type polycrystalline SnSe materials. Such thermoelectric performance makes this system highly suitable for medium‐temperature power generation. Additionally, the SnSe material exhibits enhanced mechanical properties due to solution strengthening (GeTe doping) and diffusion strengthening (Ag_2_Se precipitation). This work demonstrates that chemical bond tailoring is a universal strategy for optimizing thermoelectric performance, applicable to other material systems.

## Author Contributions

**Xing Yang**: investigation, writing – original draft, writing – review and editing, methodology, software, formal analysis. **Wang‐Qi Bao**: investigation, methodology. **Zi‐Yuan Wang**: investigation, software. **Ze Li**: investigation, software, validation. **Rong Li**: investigation. **Yi‐Xin Zhang**: funding acquisition, investigation. **Jing Feng**: funding acquisition, writing – original draft, investigation, writing – review and editing, methodology. **Zhen‐Hua Ge**: writing – original draft, investigation, funding acquisition, conceptualization, methodology, writing – review and editing. **Li‐Dong Zhao**: writing – original draft, funding acquisition, writing – review and editing.

## Conflicts of Interest

The authors declare no conflicts of interest.

## Supporting information




**Supporting File**: advs78128‐sup‐0001‐SuppMat.docx.

## Data Availability

The data that support the findings of this study are available from the corresponding author upon reasonable request.
