# Supplementary material for: Excellent Thermoelectric Performance Realized in SnSe Materials via Chemical Bond Tailoring Using GeTe as Molecule Scissors
Source: Adv Sci (Weinh). 2026 Sep 30:e78128. Online ahead of print. doi: 10.1002/advs.78128 (PMC13626166; doi:10.1002/advs.78128)
Supplement: Supplementary file 1 [file ADVS-9999-e78128-s001.docx]

**Supporting Information**

**for**

**Excellent Thermoelectric Performance Realized in SnSe Materials via Chemical Bond Tailoring Using GeTe as Molecule Scissors**

*Xing Yang^a,b^, Wang-Qi Bao^a,b^, Zi-Yuan Wang^a,b^, Ze Li^a,b^, Rong Li^a,b^, Yi-Xin Zhang^a,b^, Jing Feng^a,b^, Zhen-Hua Ge^a,b^*, and Li-Dong Zhao^c^*.*

^a^Faculty of Materials Science and Engineering, Kunming University of Science and Technology, Kunming, 650093, China.

^b^National-Local Joint Engineering Research Center for Technology of Advanced Metallic Solidification Forming and Equipment, Kunming University of Science and Technology, Kunming 650093, China.

^c^School of Materials Science and Engineering, Beihang University, Beijing, 100191, China.

**Corresponding author: Zhen-Hua Ge, Li-Dong Zhao.*

*E-mail address: ggzhh@qq.com (Z.-H. Ge), zhaolidong@buaa.edu.cn (L.-D. Zhao).*

Figure S1. EMPA-mapping information for the A-SnSe + 0.25 wt% GeTe sample (a) BSE image, (b-f) the elements of Sn, Se, Ag, Ge, and Te, respectively.

Figure S2. FESEM images of the fractured surfaces for the pristine SnSe and A-SnSe + x wt% GeTe (x = 0, 0.125, 0.25 and 0.5) bulk samples.

Figure S3. TEM information for the A-SnSe + 0.25 wt% GeTe sample (a) low magnified image and the inserted FFT image, (b) high magnified image, (c) IFFT image for the selected area of (b), and (d-e) GPA images of (b).

Figure S4. The energy band of (a) the pristine SnSe sample, (b) Ag-doped sample, and (c) (Ag, GeTe) co-doped sample.

Figure S5. The XRD patterns of the A-SnSe + 0.25 wt% GeTe sample during the heating-cooling cycles process.

Figure S6. The temperature dependence of (a) electrical conductivity (*σ*), (b) Seebeck coefficient (S), (c) total thermal conductivity (*κ*), and (d) *ZT* values of the A-SnSe + 0.25 wt% GeTe sample during the heating-cooling cycles process.

**The detailed calculations of thermal conductivity**

The thermal transport properties of all bulk samples were characterized by measuring thermal diffusivity (*D*) using a laser flash apparatus (Netzsch LFA-457). The sample density (*ρ*) was determined by the Archimedes methos using deionized water as the immersion medium.

The total thermal conductivity (*κ*_tot_) was calculated using the following relationship: *κ*_tot_ = *D C*_p_ *ρ,* where *C*_p_ is the specific heat capacity using the Dulong-Petit law. The electron thermal conductivity (*κ*_e_) was calculated using the Wiedemann–Franz relation: *κ*_e_ = *L σ T*, where *σ* is the electrical conductivity, T is the absolute temperature, and the L is the Lorenz number. In this study, the Lorenz number was determined using the modified single parabolic band (SPB) model: *L = 1.5 + exp (– (|S|/116))*^[1]^. Finally, the lattice thermal conductivity (*κ*_l_) was obtained by subtracting the electronic contribution the total thermal conductivity: *κ*_l_ = *κ*_tot_ - *κ*_e_.

**References**

[1] H.-S. Kim, Z. M. Gibbs, Y. Tang, H. Wang and G. J. Snyder,Characterization of Lorenz number with Seebeck coefficient measurement, APL Mater. **2015**, *3,* 041506.
